# Investigating p.Ala1035Val in NPC1: New Cellular Models for Niemann–Pick Type C Disease

**DOI:** 10.3390/ijms252212186

**Published:** 2024-11-13

**Authors:** Hugo David, Jlenia Monfregola, Isaura Ribeiro, Maria Teresa Cardoso, Ana Catarina Sandiares, Luciana Moreira, Maria Francisca Coutinho, Dulce Quelhas, Andrea Ballabio, Sandra Alves, Marisa Encarnação

**Affiliations:** 1 Research and Development Unit, Department of Human Genetics, National Institute of Health Doutor Ricardo Jorge (INSA, I. P.), Rua Alexandre Herculano 321, 4000-055 Porto, Portugal; hugoddavid@hotmail.com (H.D.); ana.sandiares@insa.min-saude.pt (A.C.S.); luciana.c98@gmail.com (L.M.); francisca.coutinho@insa.min-saude.pt (M.F.C.); sandra.alves@insa.min-saude.pt (S.A.); 2 Center for the Study of Animal Science—Instituto de Ciências, Tecnologias e Agroambiente da Universidade do Porto (CECA-ICETA), University of Porto, Praça Gomes Teixeira, Apartado 55142, 4051-401 Porto, Portugal; 3 Associate Laboratory for Animal and Veterinary Sciences (AL4AnimalS), Faculdade de Medicina Veterinária, Avenida da Universidade Técnica, 1300-477 Lisboa, Portugal; 4 Telethon Institute of Genetics and Medicine (TIGEM), Via Campi Flegrei 34, 80078 Pozzuoli, Italy; j.monfregola@tigem.it (J.M.); ballabio@tigem.it (A.B.); 5 Laboratório de Bioquímica Genética, Serviço de Genética Laboratorial, Clínica de Genética e de Patologia, Centro de Genética Médica Jacinto de Magalhães, Unidade Local de Saúde de Santo António, 4099-001 Porto, Portugal; isauraribeiro.cgm@chporto.min-saude.pt (I.R.); dulce.quelhas@chporto.min-saude.pt (D.Q.); 6 Unidade Multidisciplinar de Investigação Biomédica (UMIB), Instituto de Ciências Biomédicas Abel Salazar (ICBAS), University of Porto, Rua Jorge de Viterbo Ferreira 228, 4050-313 Porto, Portugal; 7 Laboratory for Integrative and Translational Research in Population Health (ITR), Institute of Public Health, University of Porto, Rua das Taipas 135, 4050-600 Porto, Portugal; 8 Reference Center for Diagnosis and Treatment, Centro Hospitalar e Universitário de São João (CHUSJ), Alameda Prof. Hernâni Monteiro, 4200-319 Porto, Portugal; mtcpteresa@gmail.com

**Keywords:** Niemann–Pick type C, NPC1, p.Ile1061Thr, p.Ala1035Val, p.Ile858Val, cell models, ARPE-19, lysosomal storage disorders, complex alleles, phenotypic variability

## Abstract

Niemann–Pick type C (NPC) is a lysosomal storage disorder (LSD) caused by pathogenic variants in either the *NPC1* or *NPC2* genes, which encode proteins involved in the lysosomal export of unesterified cholesterol. In patients of Western European descent, the p.Ile1061Thr variant in *NPC1* is especially prevalent. However, mounting evidence has positioned p.Ala1035Val as the most common variant in Portugal and the second most prevalent variant worldwide. By analyzing 10 Portuguese NPC patients homozygous for p.Ala1035Val, we found an SNP in *cis* on position 858 (p.Ile858Val), which we hypothesize could have a disease-modifying effect. To address this query, we created variant-specific in vitro models of NPC by stably transducing *NPC1*^−/−^ ARPE-19 cells with constructs encoding different fluorescently-tagged variants of NPC1, which we used, alongside patient-derived skin fibroblasts, to investigate lysosomal positioning and the trafficking routes elicited by p.Ile1061Thr and p.Ala1035Val (with and without the p.Ile858Val SNP in *cis*). Our results corroborate the previously described decrease in p.Ile1061Thr-NPC1 trafficking to the lysosome and suggest a similar, if not worse, scenario for the p.Ala1035Val variant, especially when in *cis* with p.Ile858Val. This is the first reported functional study addressing the impact of the p.Ala1035Val variant at the cellular level, paving the way for novel therapeutic options.

## 1. Introduction

### 1.1. Niemann–Pick Type C Disease

Niemann–Pick type C (NPC, ORPHA:646) is a rare, monogenic, progressive and neurodegenerative lysosomal storage disease (LSD). Cholesterol and other lipids accumulate inside lysosomes, triggering a cascade of cellular and molecular events, culminating in the severe neurovisceral symptomatology associated with this disorder [1].

The disease is estimated to affect around 1 in every 90,000 live births worldwide [2], although this incidence rate is likely to be underestimated, due to the number of belated diagnoses and misdiagnoses of NPC.

Clinical presentations sway between a swiftly progressing neonatal form to an adult-onset chronic condition. Even though this disease is often described as somewhat of a continuum of symptomatic evolution, the most broadly accepted classification groups patients considering their age at neurological onset and their clinical manifestation timeline [3]. Ascites and hydrops fetalis are common “perinatal” presentations of NPC, emphasizing the underlying organomegaly [4]. Cases where the first neurologic symptoms become apparent before the patients reach 2 years of age are categorized as “early-infantile”, with hepatosplenomegaly and liver problems becoming nearly invariable manifestations of disease [5]. The “late-infantile” form of NPC (i.e., between 2 and 6 years old) often exacerbates (hepato)splenomegaly, and the first neurologic symptoms usually include gelastic cataplexy, ataxia, seizures, and impediments in speech and fine motor skills [6]. Patients neurologically diagnosed between 6 and 15 years old represent the “juvenile” form of disease, wherein, besides the almost ever-present hepatosplenomegaly, vertical supranuclear gaze palsy (VSGP) becomes apparent, and attention deficits and motor impairments aggravate [6]. The “adult” form of disease often includes psychiatric as well as neurologic complications, such as severe ataxia, dysarthria, dysphagia, dystonia, dementia, and psychosis, which present after the age of 15 [7].

This vast phenotypic variability, alongside the rarity of NPC, often leads to prolonged and arduous diagnostic odysseys, where clinicians struggle to identify the underlying disorder and patients suffer irreversible damage while access to therapy is delayed.

### 1.2. Pathogenesis

In over 95% of cases, the affected gene is *NPC1*, with *NPC2* being responsible for the remaining 5% [8]. However, a puzzling feature of NPC disease is that even patients with similar genetic backgrounds and displaying the same disease-causing variant can exhibit significantly different symptoms and clinical evolution.

The *NPC1* gene spans 55 kb on the reverse strand of human chromosome 18 [9]. Its cDNA sequence comprises 25 exons and predicts a 1278 amino acid protein, which is co-translationally *N*-glycosylated and folded in the endoplasmic reticulum (ER) before being transported through the Golgi apparatus where it undergoes processing to a mature complex glycosylated protein, which ultimately localizes to late endosomes/lysosomes (LE/Lys).

**Figure 1:**
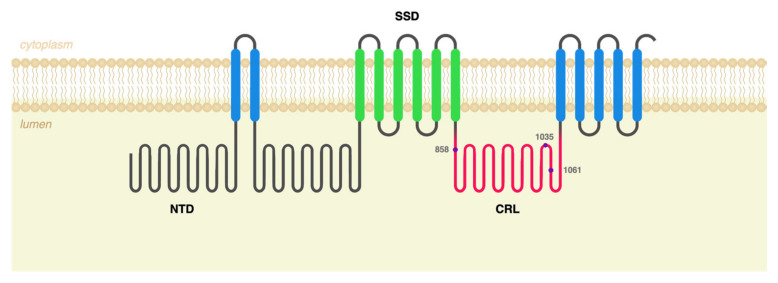
NPC1 protein topology. The human NPC1 protein has 13 transmembrane domains (blue and green), connected by 4 small and 3 large loops that protrude into the lysosomal lumen and 5 cytoplasmic loops, culminating in a C-terminal cytoplasmic domain. Cholesterol is delivered to the protein through its N-terminal domain (NTD), which hands the molecules over to its putative sterol-sensing domain (SSD, in green). Most disease-causing mutations in NCP1 are clustered within the protein’s cysteine-rich loop (CRL, in pink), which is thought to be particularly critical for normal protein function. The three variants studied in this article (in purple) all localize to the CRL.

Over 700 disease-causing variants have been identified in the *NPC1* gene, with over half of those being missense, leading to conformational modifications that result in the disruption of NPC1 trafficking [10]. A significant proportion of those missense variants are clustered within the protein’s cysteine-rich loop (CRL), but depending on the affected amino acid’s position, disease outcome can be quite variable (Figure 1).

In patients of Western European descent, the most frequently reported variant is p.Ile1061Thr (c.3182T>C) [11], and numerous research efforts have focused on its impact. When in homozygosity, it has been suggested that this variant correlates with juvenile clinical presentation.

In Portugal, the most common disease-causing variant has long been known to be p.Ala1035Val (c.3104C>T) [12]. However, recently, a large cohort study has reclassified p.Ala1035Val as the second most prevalent variant worldwide [13]. From the phenotype observed in homozygous individuals, there seems to be a correlation with infantile presentation of NPC disease. Still, there is little to no literature investigating the effects of this variant. In fact, no information is known about p.Ala1035Val-NPC1 trafficking and degradation.

### 1.3. Study Overview

Here, we started by analyzing sequencing results from 10 Portuguese patients and their parents (whenever available), and our data support that the p.Ala1035Val variant presents in *cis* with other benign variants, including a semiconservative amino acid substitution at position 858 (p.Ile858Val, c.2572A>G), whose presence was not observed in the only Portuguese patient homozygous for the p.Ile1061Thr variant, according to published data by Ribeiro et al. [12].

Taking all of these observations into account, we hypothesized that the isoleucine to valine substitution on position 858 could contribute to the exacerbation of NPC1 malfunction and subsequent lysosomal dysfunction in patients with the p.Ala1035Val variant. To test this, we leveraged a previously established *NPC1*^−/−^ ARPE-19 cell line and created new stable cell lines transduced with wildtype (WT), p.Ile1061Thr, p.Ala1035Val and p.Ala1035Val + p.Ile858Val *NPC1* variants.

Using these cells, alongside patient-derived skin fibroblasts, we surveyed the trafficking routes elicited by these variants, and our results revealed a possible disease modifying effect for the p.Ile858Val variant when in *cis* with p.Ala1035Val, indicating that this genetic combination may aggravate NPC1 retention in the ER in these patients.

In addition, these newly established cell lines may serve possible future studies aiming to understand the effect of known and novel missense *NPC1* variants or may even be used to test which of those genetic variants may be good candidates for proteostasis modulator-based approaches.

## 2. Results

### 2.1. c.3104C>T (p.Ala1035Val) Was Identified in cis with Other Polymorphisms in 10 Portuguese NPC Patients

**Figure 2:**
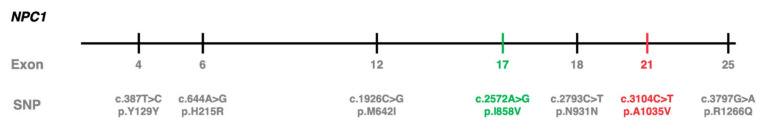
Single-nucleotide polymorphisms (SNPs) identified alongside the c.3104C>T (p.Ala1035Val) pathogenic variant in *NPC1*. Among the SNPs in linkage with the c.3104C>T (p.Ala1035Val) pathogenic variant (red), c.2572A>G (p.Ile858Val), in exon 17, is highlighted (green).

Within the scope of the molecular characterization of the *NPC1* gene for patients with high suspicion scores for NPC disease, other variants, or single-nucleotide polymorphisms (SNPs), were identified alongside the disease-causing variant, p.Ala1035Val (Figure 2). Each of those SNPs was found to be individually frequent in the population, according to data from the Genome Aggregation Database (gnomAD), but the combination of the six SNPs in *cis* with the c.3104C>T (p.Ala1035Val) variant—and not with other *NPC1* pathogenic variants—seemed worthy of further investigation.

Of note, the c.2572A>G SNP, resulting in a semiconservative amino acid substitution at position 858 (p.Ile858Val), had been previously reported to have a modifying effect on NPC disease, despite being a relatively frequent modification on its own, with a frequency of 0.49 according to gnomAD. This same SNP has also been identified in *cis* with the p.Ala1035Val variant in a reported Spanish NPC patient [14].

Another frequent SNP in the *NPC1* gene described as having an additive effect is c.2793C>T (p.Asn931=). This variant has been reported to impact the penetrance of a rare synonymous variant, leading to the activation of a cryptic splice site (SS) when both variants are present in *cis* [15]. In fact, the existence of complex alleles such as these could partially explain the phenotypic diversity for a number of LSDs, including NPC disease.

### 2.2. NPC1^−/−^ ARPE-19 Cells Retrovirally Transduced with Either WT or Mutant NPC1 Forms Mimic the Expected Cellular Phenotype

In order to address the raised hypothesis, cell lines were created to study the p.Ala1035Val variant in both the presence and absence of the p.Ile858Val SNP.

**Figure 3:**
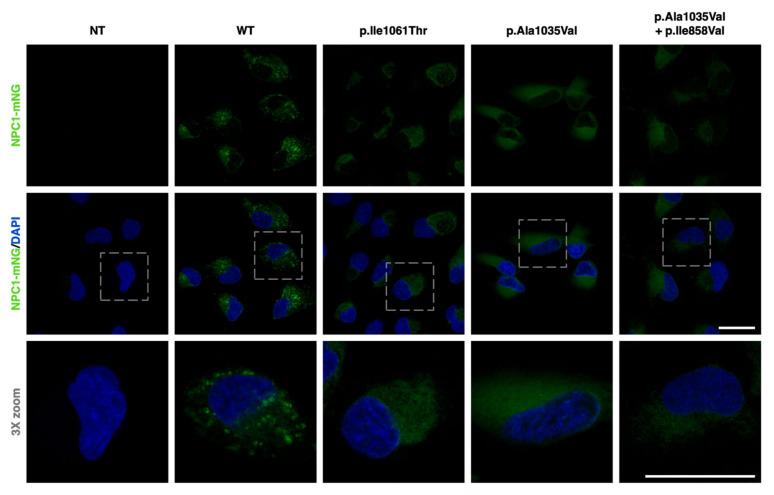
NPC1-mNeonGreen expression in non-transduced and transduced *NPC1*^−/−^ ARPE-19 cells. The top panel shows the NPC1-mNeonGreen signal (green), the middle panel displays a merging of that signal with DAPI (blue), and the bottom panel is a 3X zoom of the merged image. From left to right, each column exemplifies a condition of *NPC1*^−/−^ ARPE-19 cells; the first column shows non-transduced (NT) cells, while the rest represent cells infected with retroviral particles containing either the wildtype (WT), p.Ile1061Thr, p.Ala1035Val or p.Ala1035Val + p.Ile858Val constructs. The cells infected with the WT construct exhibit a punctate pattern consistent with its LE/Lys localization, and those infected with p.Ile1061Thr display a more diffuse pattern. These are representative images obtained through confocal fluorescence microscopy. Scale bars (white) represent 25 μm.

Genetically engineered *NPC1*^−/−^ ARPE-19 cells recapitulate the cellular pathogenic phenotype of NPC disease. Taking advantage of their ease of transduction, these cells were infected with in-house-generated γ-retroviral particles, carrying the different variants (and variant combination) under study in a pBABE-puro construct that contained the *NPC1* cDNA sequence fused with that of the mNeonGreen fluorescent protein. NPC1-mNeonGreen expression was then visualized through fluorescence microscopy (Figure 3).

Images confirmed the absence of NPC1-mNeonGreen signal in non-transduced *NPC1*^−/−^ ARPE-19 cells. Cells transduced with the WT form of NPC1-mNeonGreen displayed a punctate pattern, which was consistent with its LE/Lys localization. On the other hand, cells transduced with the p.Ile1061Thr variant exhibited a less intense and much more diffuse pattern, which was consistent with its previously described retention and degradation in the ER [16,17]. Cells expressing the p.Ala1035Val variant (with and without the p.Ile858Val SNP) also presented a dim and dispersed expression pattern, hinting at a conceivably similar targeting for degradation before reaching the LE/Lys compartment.

### 2.3. NPC1^−/−^ ARPE-19 Cells Retrovirally Transduced with the p.Ala1035Val Variant of NPC1 Display Higher Perinuclear Lysosome Clustering

Seeing as the accumulation of aberrant cholesterol within the LE/Lys compartment and the consequential juxtanuclear clustering of lysosomes is an important cellular hallmark of NPC disease [18,19], lysosomal positioning was surveyed in the *NPC1*^−/−^ ARPE-19 cell lines transduced with the different variants (and variant combinations) of *NPC1*.

**Figure 4:**
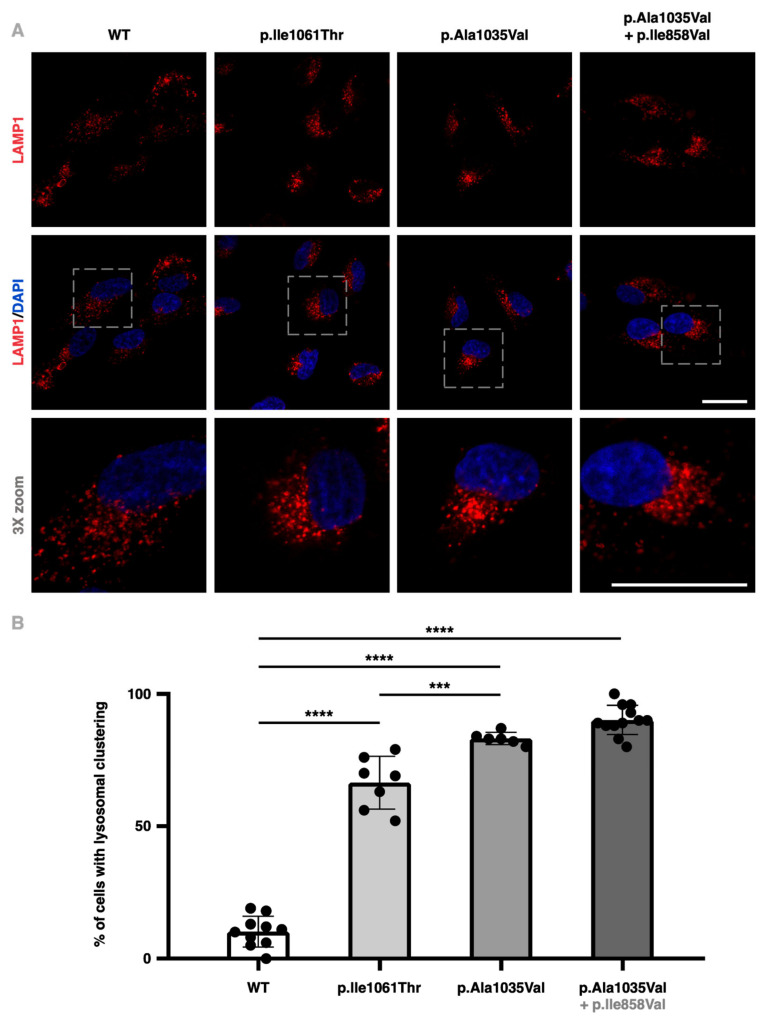
Perinuclear lysosome clustering in transduced *NPC1*^−/−^ ARPE-19 cells. (**A**): The top panel shows the LAMP1 signal (red), the middle panel displays a merging of that signal with DAPI (blue), and the bottom panel represents a 3X zoom of the merged image. From left to right, each column exemplifies a condition of *NPC1*^−/−^ ARPE-19 cells infected with retroviral particles carrying either the wildtype (WT), p.Ile1061Thr, p.Ala1035Val or p.Ala1035Val + p.Ile858Val constructs. These are representative images obtained through confocal fluorescence microscopy. Scale bars (white) represent 25 μm. (**B**): Scoring of lysosome clustering in transduced *NPC1*^−/−^ ARPE-19 cells as a percentage of cells presenting a clustering pattern for each condition. At least six fields per condition were randomly selected for scoring, comprising over 300 cells per condition. Comparisons between groups were analyzed using a one-way analysis of variance significance analysis (*** *p* < 0.001; **** *p* < 0.0001).

Through immunofluorescence labelling of the lysosomal-associated membrane protein 1 (LAMP1)—a type I transmembrane glycoprotein expressed across lysosomal membranes—significant accumulation and clustering of lysosomes were observed in the perinuclear region of *NPC1*^−/−^ ARPE-19 cells transduced with the mutant *NPC1* constructs (Figure 4A). Quantification was performed by visually inspecting and scoring the clustering of lysosomes in images from different fields in an unbiased or blinded manner, as previously described [20,21].

Results revealed an increase from 10.27 (±1.85)% in cells transduced with the WT form of the protein to 66.27 (±3.82)% in those expressing the p.Ile1061Thr variant, 83.13 (±0.97)% in cells transduced with the p.Ala1035Val variant alone, and 90.33 (±1.59)% for cells transduced with p.Ala1035Val in *cis* with p.Ile858Val (Figure 4B). These results are consistent with the increased LAMP1 protein detection observed through Western blotting in patient-derived skin fibroblasts and are in accordance with the literature [22].

### 2.4. p.Ala1035Val-NPC1 and p.Il11061Thr-NPC1 Are More Endo-H-Sensitive than WT-NPC1

While many studies concerning the intracellular trafficking of p.Ile1061Thr-NPC1 have been conducted, no such efforts have been reported for the p.Ala1035Val variant.

Here, Endoglycosidase H (Endo H) sensitivity was used to evaluate mutant NPC1 trafficking through the medial Golgi in patient-derived skin fibroblasts. As NPC1 traffics through the Golgi apparatus, its *N*-linked glycans are processed and mature as their mannose residues are progressively replaced with other sugar monomers, turning WT-NPC1 into a complex glycosylated protein; it thus becomes resistant to cleavage by the Endo H enzyme, which is only able to target high-mannose asparagine-linked oligosaccharides from simple to hybrid glycoproteins, as opposed to Peptide-*N*-glycosidase F (PNGase F), which cleaves virtually all *N*-linked glycans from simple to complex glycoproteins, which here were used as a positive control.

**Figure 5:**
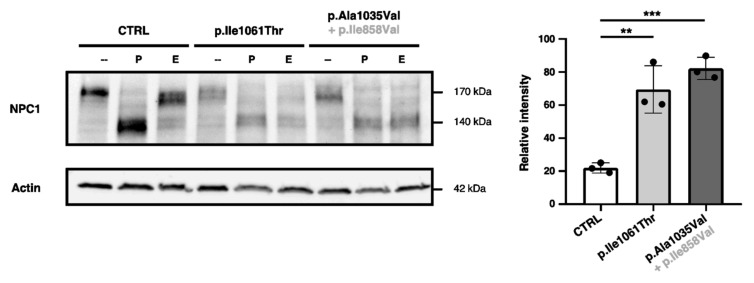
Endo H sensitivity quantification in different NPC1 variants. The left panel displays a representative Western blot showing both glycosylated and deglycosylated NPC1 protein levels in a human skin fibroblast cell line used as a control (CTRL) and skin fibroblasts obtained from NPC patients with the p.Ile1061Thr and p.Ala1035Val (and p.Ile858Val) variants in NPC1, each incubated with no treatment (-), PNGase F (P), or Endo H (E) and Actin as a loading control. The right panel displays the quantification of the deglycosylated (Endo H-sensitive) NPC1 protein–total NPC1 protein, for three independent experiments (*n* = 3). Comparisons between groups were analyzed using a one-way analysis of variance to assess significance (** *p* < 0.01; *** *p* < 0.001).

In control (CTRL) skin fibroblasts, only about 21.19 (±1.01)% of NPC1 protein displayed Endo H sensitivity, while human p.Ile1061Thr-NPC1 and p.Ala1035Val-NPC1 both exhibited around 3.50-fold increases in Endo H sensitivity when compared to the control, with levels around 69.46 (±4.79)% for the p.Ile1061Thr variant and 82.19 (±2.26)% for p.Ala1035Val (Figure 5). These data support that these mutant forms of the NPC1 protein are retained before reaching the Golgi, never maturing into its fully mature status.

### 2.5. NPC1^−/−^ ARPE-19 Cells Retrovirally Transduced with the p.Ala1035Val + p.Ile858Val Variant Combination of NPC1 Display Attenuated LAMP1 Colocalization

**Figure 6:**
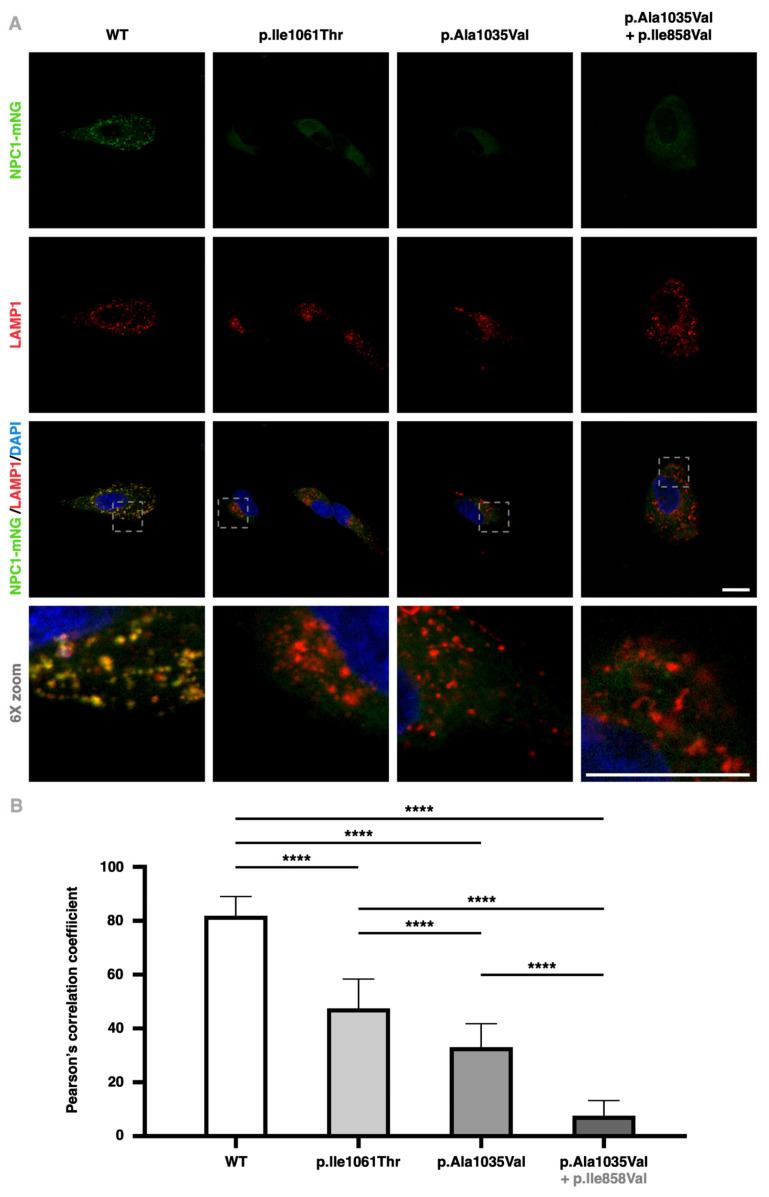
LAMP1 and NPC1-mNeonGreen cross colocalization in transduced *NPC1*^−/−^ cells. (**A**): The first line shows the NPC1-mNeonGreen signal (green), the second line represents the LAMP1 signal (red), the third line displays a merging of both those signals as well as DAPI (blue), and the bottom line represents a 6X zoom of the merged image. From left to right, each column exemplifies a condition of NPC1^−/−^ ARPE-19 cells infected with either the wildtype (WT), p.Ile1061Thr, p.Ala1035Val, or p.Ala1035Val + p.Ile858Val constructs. These are representative images, obtained through confocal fluorescence microscopy. Scale bars (white) represent 15 μm. (**B**): Pearson’s correlation coefficient for each of at least 50 unbiasedly selected regions of interest analyzed for each cell line transduced with the different variants of *NPC1*. Comparisons between groups were analyzed using a one-way analysis of variance significance analysis followed by Tukey’s test (**** *p* < 0.0001).

Immunofluorescence assays on *NPC1*^−/−^ ARPE-19 cells infected with the different *NPC1* constructs substantiate that, unlike the WT protein, the p.Ile1061Thr and p.Ala1035Val variants fail to traffic through the Golgi, being retained in the ER, failing to colocalize with the LAMP1 protein (Figure 6A).

Cells expressing the native form of the NPC1 protein display a close to total colocalization of NPC1-mNeonGreen with LE/Lys vesicles (LAMP1-positive organelles), with an average Pearson’s correlation coefficient of 0.82 (±0.01). Mutant NPC1 proteins presented progressively attenuated LAMP1 colocalization figures of 0.47 (±0.01) for p.Ile1061Thr, 0.33 (±0.01) for p.Ala1035Val alone, and 0.07 (±0.01) for p.Ala1035Val in *cis* with p.Ile858Val (Figure 6B).

## 3. Discussion

Despite first being described over 100 years ago, many aspects of NPC disease are still quite enigmatic to this day, namely the vast phenotypic variability observed amongst patients. Of the many factors that may influence the phenotypic manifestation of this disease, we postulate that the presence of the p.Ile858Val SNP in patients homozygous for the p.Ala1035Val variant in *NPC1* could augment the severity of NPC disease. This possibility was initially raised in a study on 12 unrelated Caucasian NPC patients, which suggested that the allelic variant c.2572G (Val858) was associated with the NPC phenotype. In said patient group, 72.7% of *NPC1* alleles contained the 2572G allele compared to only 40.6% in the control group. Notably, in three of the four cases where only one pathogenic mutation was identified, the haplotype with the “missing” variant also contained the 2572G allele. This observation suggested a potential modifying effect of this semiconservative amino acid change (or its underlying haplotype) with the substitution from an isoleucine to a valine at position 858, potentially influencing the expression of pathogenic missense variants in *NPC1* [23], although no functional studies were performed at the time. Moreover, both p.Ile858Val and p.Ala1035Val are located in the conserved cysteine-rich luminal domain [24]. Given the amount of individuals homozygous for p.Ile858Val reported on the gnomAD database, the p.Ile858Val variant alone does not appear to have a pathogenic effect on NPC1 but might influence the expression of other *NPC1* pathogenic variants. Whilst the NPC1 protein is very sensitive to genetic perturbation and p.Ala1035Val itself already dictates a deleterious effect, this complex allele in *NPC1*—for which no functional studies have been published—could exacerbate the disease phenotype. To corroborate this hypothesis, more patients need to be recruited and analyzed in clinical aspects to understand if the disease’s evolution and lifespan vary amongst those with p.Ala1035Val alone (if there are any) and those with the complex allele (p.Ile858Val + p.Ala1035Val), like the patients considered in this study.

Since all the patients homozygous for the p.Ala1035Val variant included in our cohort also presented the p.Ile858Val SNP in *cis*, we were not able to study the p.Ala1035Val variant alone using their skin fibroblasts. To circumvent this limitation, we made use of a previously established *NPC1*^−/−^ ARPE-19 cell line—which, unlike skin fibroblasts, recapitulate several features of the pathological cellular phenotype of NPC—and transduced it with retroviral particles loaded with different constructs encoding different WT and mutant variants of *NPC1*.

Using these newly created variant-specific disease models, alongside patient-derived skin fibroblasts, we examined several important mechanisms involved in NPC pathogenesis and revealed a possible disease-modifying effect for the p.Ile858Val variant, which was found in *cis* in all the analyzed Portuguese p.Ala1035Val alleles.

Our data collectively suggest that the presence of the p.Ala1035Val variant, especially when combined with the p.Ile858Val SNP in *cis*, leads to altered LE/Lys positioning and a dramatic decrease in NPC1 trafficking to the lysosome. This implies that patients carrying p.Ala1035Val—one of the most prevalent and severe *NPC1* pathogenic variants worldwide—might benefit from proteostasis modulator-based approaches.

In fact, *NPC1* variants have emerged as latent targets for proteostasis modulators, but drug development efforts have so far been unsuccessful in mouse models. The issue may be that such models are inadequate for the translational study of NPC1 protein trafficking and degradation.

Taking that into account, we set out to provide an alternative to mouse or fibroblast models by developing new variant-specific models of disease, which accurately mimic the NPC disease phenotype. In the future, we hope that the stable cell lines we created may enable the study of variant-specific trafficking patterns and even large-scale drug screenings, thus facilitating novel drug discovery (or repurposing) and potentiating the number of patients eligible for its benefits.

## 4. Materials and Methods

### 4.1. Cell Lines

Three primary human dermal fibroblast cell lines were used as controls in several experiments. One adult human dermal fibroblast (HDFa) cell line (P10856) was commercially acquired from Innoprot (Derio, Spain). The other two were pseudoanonymized primary fibroblast cell lines already available at the lab. This study was conducted in accordance with the Declaration of Helsinki and approved by the Ethics Committee of the National Institute of Health Dr. Ricardo Jorge, INSA, I.P. (dates of approval: 17 November 2020, 18 April 2023 and 16 May 2023).

For the production of γ-retroviral particles carrying the different *NPC1* constructs, a human embryonic kidney 293 T (HEK293T) cell line was used, having been kindly gifted by John Neidhart at the Carl von Ossietzky University of Oldenburg (Oldenburg, Germany).

ARPE-19 and *NPC1*^−/−^ ARPE-19 cells were kindly gifted through Andrea Ballabio’s group at the Telethon Institute of Genetics and Medicine (Pozzuoli, Italy).

### 4.2. Plasmids

pBABE-puro + NPC1-mNeonGreen was a kind gift from Kartik Chandran. pCMV-VSV-G and pUMVC were both gifts from Bob Weinberg (Addgene plasmids #8454 and #8449, respectively).

### 4.3. Antibodies

The following primary antibodies were used [antibody, dilution (vendor, reference)]: mouse α-Actin(C-2), 1:5000 (sc-8432, Santa Cruz Biotechnology, Dallas, TX, USA); mouse α-LAMP1(H4A3), 1:500 Western and 1:200 imaging (sc-20011, Santa Cruz Biotechnology, Dallas, TX, USA); and rabbit α-NPC1, 1:5000 (a134113, Abcam, Cambridge, UK).

As for secondary antibodies, the ones used were the following [antibody, dilution (vendor, reference)]: goat α-mouse IgG-HRP, 1:40,000 (sc-2005, Santa Cruz Biotechnology, Dallas, TX, USA); mouse α-rabbit IgG-HRP, 1:40,000 (sc-2357, Santa Cruz Biotechnology, Dallas, TX, USA); and AlexaFluor^TM^ 594 goat α-mouse IgG(H + L), 1:500 (A-11032, Invitrogen, Waltham, MA, USA).

### 4.4. Site-Directed Mutagenesis

The QuikChange Lightning Site-Directed Mutagenesis Kit (Agilent Technologies, Santa Clara, CA, USA) was chosen to insert the p.Ile1061Thr, p.Ala1035Val, and p.Ile858Val variants into the pBABE-puro + NPC1-mNeonGreen expression vector using pairs of complementary oligonucleotide primers containing each desired mutation, according to the manufacturer’s instructions. The success of each mutagenesis was confirmed by sequencing.

### 4.5. Generation of Stably Transduced NPC1^−/−^ ARPE-19 Cells

HEK293T cells were grown to 70% confluency in 12-well tissue-culture-treated plates before being transfected with the different transfer (pBABE-puro + NPC1-mNeonGreen), packaging (pUMVC) and envelope (pCMV-VSV-G) plasmid DNA mixes, following a 3:2:1 ratio. Subsequently, 10 μL of the appropriate 1 μg/μL plasmid DNA mix was added to 37 μL of room-temperature Opti-MEM medium (Gibco^TM^, Grand Island, NY, USA), before vortexing and adding in 3 μL of room-temperature FuGENE^®^ HD transfection reagent (Promega, Madison, WI, USA). The different mixes were incubated for 5 to 15 min at room temperature, before adding them, dropwise, into the corresponding well. At 16 h post-transfection, 1 mL viral collection medium (VCM; 20 μM HEPES in DMEM supplemented with 10% FBS) was added to each well. Virus-rich medium (VRM) was collected in a Biosafety Level 3 laboratory at 32 h and 56 h after adding the VCM and filtered through a 0.45 μm filter.

For the retroviral transduction, *NPC1*^−/−^ ARPE-19 cells were grown on 6-well plates to approximately 80% confluency and transduced using 500 μL of filtered VRM mixed with 500 μL DMEM/F-12 (Gibco^TM^, Grand Island, NY, USA) with 16 μg/mL polybrene (the final concentration in the wells was 8 μg/mL). Transduced cell populations were selected using 1 μg/mL puromycin, and the medium was replaced every 48 h.

### 4.6. Immunofluorescence Assays

Once the retrovirally transduced cells reached the proper confluency, they were seeded in 8-well Lab-Tek II chamber slides (Nunc^TM^, Roskilde, Denmark) for subsequent immunofluorescence assays, processed as previously described [25,26].

### 4.7. Enzymatic Deglycosylation and Western Blot

Following the protocol detailed by Schultz et al. in 2022 [16], dermal fibroblasts and ARPE-19 cells were pelleted, lysed, and digested using Endoglycosidase H (Endo H) and Peptide-N-Glycosidase F (PNGase F) enzymes (New England BioLabs^®^, Ipswich, MA, USA).

To visualize NPC1, 13 μg of total protein was mixed with NuPAGE^TM^ LDS Sample Buffer and NuPAGE^TM^ Sample Reducing Agent (Invitrogen, Waltham, MA, USA) and denatured at 70 °C for 10 min before loading it onto Mini-PROTEAN^®^ Stain-free 4–15% precast gels (Bio-Rad, Hercules, CA, USA), which ran for 45 min at 200 V. The stratified proteins were then transferred to a PROTRAN^®^ nitrocellulose membrane (Whatman^®^, Maidstone, UK), using a 300 mA current for 45 min, in a Mini-PROTEAN^®^ Tetra System (Bio-Rad). The membrane was blocked for 1 h using 5% BSA before an overnight incubation at 4 °C with the primary antibodies. The next day, membranes were rinsed with PBST 3 × 15 min and incubated with the respective secondary antibodies for 40 min. After 3 new 15 min rinses with PBST, immunoreactivity was detected on a ChemiDoc^TM^ XRS+ imaging system (Bio-Rad, Hercules, CA, USA) following 5 min incubation with the Clarity^TM^ Western ECL Substrate (Bio-Rad, Hercules, CA, USA).

### 4.8. Lysosome Clustering Scoring

Perinuclear clustering of lysosomes in transduced *NPC1*^−/−^ ARPE cells was scored in an unbiased manner by observing the LAMP1 immunostained slides under a Leica (Wetzlar, Germany) DM4000 B fluorescence microscope, with its 40X dry objective, and selecting various random fields and counting cells with and without a clustering pattern (more than 300 cells were analyzed for each condition). Finally, the number of cells displaying lysosomal clustering was divided by the total number of cells analyzed for each condition to obtain the ratio of this phenomenon for each variant.

### 4.9. Colocalization Quantification

Over 50 regions of interest were selected for each condition of differently transduced *NPC1*^−/−^ ARPE-19 cells, and the LAMP1 and NPC1-mNeonGreen signals’ cross correlation was determined using the Coloc2 plugin within Fiji software (version 2.9.0, open source), thus obtaining the Pearson’s correlation coefficient for each region of interest.

### 4.10. Image Processing

Fluorescence microscopy images were processed using LAS X (Leica, Wetzlar, Germany) and Fiji (version 2.9.0, open source) software. Imaged membranes were handled using Image Lab software (version 6.1, Bio-Rad, Hercules, CA, USA).

### 4.11. Statistical Analysis

Statistical analysis was performed using GraphPad Prism software (version 8.0.1, Boston, MA, USA), and to analyze significance, we used a one-way analysis of variance followed by Tukey’s multiple comparisons test.

## Data Availability

The original contributions presented in the study are included in the article; further inquiries can be directed to the corresponding author.
